# Improvements in operant memory of *Aplysia* are correlated with age and specific gene expression

**DOI:** 10.3389/fnbeh.2023.1221794

**Published:** 2023-10-23

**Authors:** Eric C. Randolph, Lynne A. Fieber

**Affiliations:** Department of Marine Biology and Ecology, University of Miami Rosenstiel School, Miami, FL, United States

**Keywords:** marine model, invertebrate, mollusk, neuron, transcriptomics, long term potentiation

## Abstract

The transcription factor *Aplysia* CCAAT/enhancer binding protein (*ApC/EBP*) is expressed as an immediate early gene in the cAMP responsive element binding protein (CREB) mediated gene cascade, and it has essential functions in the synaptic consolidation of memory following a learning event. Synaptic consolidation primarily involves morphological changes at neuronal synapses, which are facilitated through the reorganization of the actin and microtubular cytoarchitecture of the cell. During early nervous system development, the transmembrane synaptic protein teneurin acts directly upon neuronal presynaptic microtubules and postsynaptic spectrin-based cytoskeletons to facilitate the creation of new synapses. It is reasonable to hypothesize that *teneurin* may also be linked to learning-induced synaptic changes and is a potential candidate to be a later gene expressed in the CREB-mediated gene cascade downstream of *ApC/EBP*. To assess the role of *ApC/EBP* and *teneurin* in learning and memory in the marine snail *Aplysia californica*, young (age 7–8 months) and aged (age 13–15 months; aging stage AII) siblings of Aplysia were trained in an operant conditioning paradigm—learning food is inedible (LFI)—over 2 days, during which they learned to modify the feeding reflex. Aged *Aplysia* had enhanced performance of the LFI task on the second day than younger siblings although far more aged animals were excluded from the analysis because of the initial failure in learning to recognize the inedible probe. After 2 days of training, *ApC/EBP isoform X1* mRNA and *teneurin* mRNA were quantified in selected neurons of the buccal ganglia, the locus of neural circuits in LFI. *Teneurin* expression was elevated in aged *Aplysia* compared to young siblings regardless of training. *ApC/EBP isoform X1* expression was significantly higher in untrained aged animals than in untrained young siblings but decreased in trained aged animals compared to untrained aged animals. Elevated levels of *ApC/EBP isoform X1* and *teneurin* mRNA before training may have contributed to the enhancement of LFI performance in the aged animals that successfully learned.

## Introduction

Consolidation of experience into long-term memory (LTM) requires RNA transcription, protein synthesis, and the transport of RNA and proteins to the synapse to either create a new synapse or effect changes in the existing ones (Goelet et al., 1986; Bailey and Chen, 1988a,b; Bailey and Kandel, 2008). In *Aplysia californica* (Aplysia), consolidation and the stable long-term synaptic changes that accompany it are achieved by the regulated expression of many genes. Of note are *CREB* and *ApC/EBP* (Alberini and Kandel, 2015), which encode transcription factors (Alberini, 2009a) that then regulate the expression of effector genes necessary for the modulation of neuronal synapses (Alberini, 2009b). Regulated gene expression is also necessary for memory reconsolidation after recall (Alberini, 2009b; Alberini and Kandel, 2015), as well as new protein synthesis at the site of previously altered synapses (Cai et al., 2012; Lee et al., 2012).

The neural circuit controlling the feeding reflex of *Aplysia* has been mapped and studied well (Cropper et al., 2004). This circuit contains neurons from both the buccal and cerebral ganglia. Previous studies have discovered molecular and morphological changes in this circuit, attributed to learning that food is inedible (LFI). These include induction of *ApC/EBP* in buccal ganglia mechanoafferent neurons that detect and transduce information about touch (Levitan et al., 2008, 2012), and changes in the number of synaptic connections to motoneurons (Tam et al., 2020), as well as in cerebral-buccal interneurons in the form of decreased responsiveness to acetylcholine (McManus et al., 2019). No such gene expression signatures have been recorded in the buccal ganglia motoneurons. Here, we studied gene expression in buccal ganglia neurons without the contribution of the sensory buccal S cluster (BSC) neurons in a test of the hypothesis posed by Levitan et al. (2012) that the CREB and C/EBP learning cascade may be key to formation of the memory of LFI. The case we studied was the next-day reconsolidated memory of LFI, after evidence that LFI occurred.

Most LFI studies focus on changes in presynaptic sensory and interneuron connections to motoneurons to explain behavioral changes even though both presynaptic and postsynaptic changes contribute to learning (Nargeot, 2001; Roberts and Glanzman, 2003; Momohara et al., 2022). Central pattern generator (CPG) neurons B31/B32 activate buccal ganglia motoneurons that innervate the I2 radular protraction muscle (Hurwitz et al., 1996, 2003, 2008). Since LFI results in a reduction of radular protractions and ingestion attempts in response to inedible food (Susswein et al., 1986), it was hypothesized that gene expression in buccal ganglia that includes the somas of neurons B31/B32 drives long-term synaptic changes after LFI training that result in reduced activity. To test this, targeted gene expression analysis was conducted on a subset of neurons from the buccal ganglia which included motoneurons B31/B32. *ApC/EBP isoform X1* and *teneurin* expression were quantitated from this subset of neurons for their possible involvement in LFI reconsolidation.

Several studies suggest that increased expression of *C/EBP* might occur during reconsolidation. Levitan et al. (2012) cited evidence that the increased expression of *C/EBPß* in the mammalian brain, as in Aplysia, is necessary for LTM formation during consolidation (Taubenfeld et al., 2001) with the same *C/EBPß* isoform expression necessary for reconsolidation in a different part of the brain (Milekic et al., 2007). This suggests that an increased expression of *ApC/EBP* might occur during reconsolidation of LFI in neurons other than the sensory neurons of the buccal ganglia. Additionally, Hatakeyama et al. (2004) showed that *LymC/EBP* expression in the right pedal dorsal 1 (RPeD1) neuron of *Lymnaea stagnalis* is required for the reconsolidation of operant conditioning LTM in CPG neurons. Thus, ApC/EBP expression changes during the reconsolidation of LFI in postsynaptic neurons of the buccal ganglia were investigated.

*ApC/EBP isoform X1*, previously shown to behave identically to the more commonly studied *ApC/EBP* (Lee et al., 2001), was studied because highly specific primers that spanned an exon junction were possible. *Teneurin*, which is most studied in the developing nervous system has been implicated in all aspects of synaptogenesis (Sanes and Zipursky, 2020). After learning, synaptogenesis promotes memory consolidation, which involves axon guidance, synaptic partner matching, and the organization of components at the new synapse, in which teneurin plays a critical role during development (Hong et al., 2012; Mosca et al., 2012; Mosca, 2015; Vysokov et al., 2018; DePew et al., 2019; Toro et al., 2020). Teneurin protein and transcripts have also been shown to be elevated in activated astrocytes and in the cerebral cortex, respectively, of adult rats after central nervous system (CNS) injury, indicating a putative function in CNS repair (Tessarin et al., 2019). It is possible that teneurin is involved in other functions of synaptic plasticity such as memory consolidation/reconsolidation. Therefore, *teneurin* was investigated to learn whether a change in expression of this gene during reconsolidation may be driven by the CREB and C/EBP learning cascade. Gene expression was measured 2 h after testing for LFI recall.

Since learning capabilities in LFI may change with animal age and confound gene expression, we studied sibling animals at two distinct time periods: just prior to first sexual maturity and in aging stage AII (Kempsell and Fieber, 2014). To ensure changes in performance and gene expression were a result of LFI training and not some other factor, we selected only animals that learned in LFI and demonstrated LTM of LFI for comparative analyses.

## Materials and methods

### Animal husbandry and training

A cohort of *Aplysia* was raised in the National Resource for *Aplysia* at the Rosenstiel School at the University of Miami. The animals were fed an *ad libitum* mixed diet of *Agardhiella subulata*, the red alga normally fed at the resource, romaine lettuce, and *Ulva lactuca*, a green alga, readily taken by *Aplysia* that can be folded tightly for use in the food probes used as training tools in this experiment. The animals were reared in exercise regimes, in which seawater forcefully flowed into their aquaria every 5–7 min, which caused the animals to secure themselves to the substrate or be swept up in the turbulent flow as described in Fieber et al. (2018). This mimicked the animal’s natural habitat of the intertidal rocky shores of California and provided constant neural stimulation throughout the animal’s life. Examples abound on the benefits of rearing laboratory animals in enriched environments, demonstrating that stimulation increases neural connections compared to rearing in desolate conditions (West and Greenough, 1972; Turner and Greenough, 1985; Jones and Greenough, 1996). Additionally, enriched-environment animals have been shown to perform better in cognitive tests (Kobayashi et al., 2002).

Animals were taught to recognize an inedible food source in the training protocol LFI that was adapted from Susswein et al. (1986) study. Animals were trained for 2 consecutive days. They were fasted for 48 h before testing began on Day 1 and were placed individually in 50 cm × 50 cm × 25 cm plastic aquaria containing 8L of aerated seawater, one animal per aquarium. A probe consisting of *U. lactuca* wrapped in 75-micron Nitex netting was held in the jaws of a plastic hemostat, and netted algae were presented to the animal by holding the probe approximately 1 cm in front of the animal’s oral tentacles. An animal’s voluntary stimulation of its lips by moving against the probe initiated the feeding reflex that is inherent to LFI (Susswein et al., 1986).

The animal’s behavior was noted while using a pair of stopwatches monitored by an assistant that recorded behavior times and total elapsed time. Data recorded were the latency between when the probe was first offered and when the animal took it into its mouth, the time at which the probe entered the animal’s mouth, how long the probe stayed in the animal’s mouth each time it entered, and the time at which the animal ejected the probe from its mouth. Radula scrapes were recorded when felt by the experimenter as a vibration of the hemostat and tended to accompany swallowing attempts. Training continued until a time of 3 min since the probe last exited the animal’s mouth when training of that animal ended for that day. The total time in the mouth (TTIM) was then calculated by adding together the elapsed times the probe remained in the animal’s mouth for each ingestion attempt. If TTIM was greater than 100 s on Day 1 training, the animal was regarded as successfully trained and was returned to a holding cage containing a non-experimental companion animal overnight. Animals that did not attain a TTIM greater than 100 s on Day 1 training were given the label “dud” and not used in any subsequent training. The minimum required TTIM of 100 s followed previously published protocols (Levitan et al., 2008, 2012) and was chosen to stress the importance of the animal’s failed attempts at swallowing the food probe that is necessary to establish the LTM of LFI (Katzoff et al., 2006). Day 2 training followed 26 h after Day 1 training and used the same protocol.

The percentage of total time saved relearning inedibility on Day 2 compared to Day 1 (%SAV) was calculated using the following equation:


%SAV=(1-D⁢a⁢y⁢ 2⁢T⁢T⁢I⁢MD⁢a⁢y⁢ 1⁢T⁢T⁢I⁢M)×100

If TTIM on Day 2 training was less than TTIM on Day 1, it was assumed the animal had retained memory of the previous Day 1’s training and had learned that the food was inedible (positive %SAV; +%SAV). Animals that successfully trained on Day 1 but then attained a higher TTIM on Day 2 were characterized as non-learning individuals (non-learners). These animals earned negative %SAV (-%SAV).

The occurrence of +%SAV was recorded as evidence that an animal had learned (Schwarz et al., 1991; Levitan et al., 2012). Animals with +%SAV were placed back into a holding cage for 2 h following Day 2 training and then anesthetized and dissected. Statistical analysis of learning was performed on the subset of all animals that were successfully trained in LFI and had a +%SAV.

Learning food is inedible training began in animals of the cohort at the age of 7 months. This age was chosen to precede sexual maturity in the stages of aging (Kempsell and Fieber, 2014), so that animals would be focused on feeding and not on mating; these animals were designated young in the data that follows. They had been reared in exercise for 4 months. Training in young animals occurred over the course of 42 days with groups of approximately six animals undergoing training at a time, and those achieving +%SAV were subsequently sacrificed to collect the ganglia. Young untrained control animals were sacrificed alongside their LFI-trained siblings. To test the effects of age on LFI, training and ganglia collection were repeated in sibling animals at the age of 13 months when animals were in aging stage AII (Kempsell and Fieber, 2014). Aged animals were in exercise for 9 months, and their training continued over the course of 93 days. Aged untrained control animals were sacrificed alongside their LFI-trained siblings.

A far greater number of aged animals were trained compared to their young siblings to attain a comparable sample size of +%SAV animals. Twenty young animals and fifty aged animals were trained in LFI. In the study, 10% of young and 36% of aged siblings did not attain 100 s in the mouth during Day 1 training (duds) and were excluded from further analysis. A -%SAV, where Day 2 TTIM exceeded Day 1’s TTIM of >100 s, occurred in a separate 17% and 31% of young and aged siblings, respectively. As a result, the subset of animals with +%SAV used for comparisons was 15 young and 22 aged animals for behavioral comparisons, and 7 young and 18 aged animals for gene expression comparisons because some of the young samples were lost during the RNA extraction process.

Untrained control sibling animals were reared at the same time and under the same conditions as the LFI-trained animals. Untrained control animals were not trained in LFI, so there are no behavioral data to report on these animals; however, they were used in gene expression analyses. Untrained controls fasted for 76 h before sacrifice to conform to the feeding conditions of LFI-trained animals.

The *Aplysia* stages of aging were previously established in Kempsell and Fieber (2014) and were determined in this study by the onset of sexual maturity at the age of 10 months as well as the eventual significant slowing of the righting and tail withdrawal reflexes at the age of 12 months. No fewer than 30 animals were tested in the righting reflex, and no fewer than 17 animals were tested in the tail withdrawal reflex each month. Animals tested in reflex behaviors were selected at random from the cohort. Therefore, the results include reflex times from animals that were later trained in LFI and those that remained untrained and were used as controls.

### Quantitative gene expression

**FIGURE 1:**
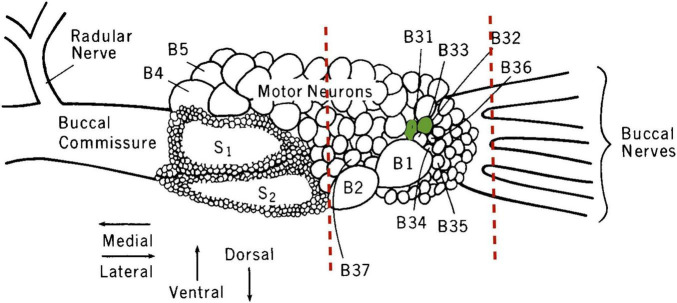
Illustration of the right buccal hemiganglion. The sites where buccal ganglia elements were trimmed away to form the B31/B32-inclusive fragment used for gene expression analysis are indicated by the dashed red lines. Neurons B31/B32 are colored green. S1 and S2 denote the buccal S cluster neurons excluded from the preparation. Modified from Susswein and Byrne (1988), with permission from Journal of Neuroscience © 1988 Society for Neuroscience.

After a 76 h fast and 2 h after Day 2 training, untrained animals or LFI-trained animals were anesthetized by injecting chilled isotonic magnesium chloride at a volume of 1/6th of their body weight into their posterior sinus. The buccal ganglia were then removed under a dissecting microscope, and conspicuous neurons B2 and B1 of the right hemiganglion were located and used as a reference for the location of nearby target neurons B31/B32 (Figure 1). A straight line cut was made next to neuron B2, and only the section of the hemiganglion containing B31/B32 minus the connectives was saved. This trimmed buccal ganglia preparation eliminated from gene expression analysis the buccal S clusters and many moto- and interneurons. Previous studies demonstrated no changes in *ApC/EBP* expression after LFI when testing buccal ganglia without BSCs (Levitan et al., 2012); thus, additional neurons were trimmed to attempt to boost the signal from moto- and interneurons associated with the feeding reflex such as B20, B51, B52, and B34. A single experimenter performed all dissections to attempt to make them as uniform as possible.

Trimmed buccal ganglia samples were placed in 1.5 mL microcentrifuge tubes containing 300 μL RNAProtect. RNA was extracted using the RNeasy Micro Kit (Qiagen RNeasy Micro Kit). The concentration and purity of RNA were determined by Nanodrop analysis of 1.5 μL of RNA (NanoDrop Technologies Model ND-1000). A total of 100 ng RNA was reverse-transcribed into cDNA using SuperScript III (Invitrogen SuperScript III First-Strand).

*ApC/EBP isoform X1*, previously shown to behave identically to the more commonly studied *ApC/EBP* (Lee et al., 2001), was studied because highly specific primers were produced that spanned an exon junction. This was impossible for *ApC/EBP* as its gene is intronless and using previously published primers for *ApC/EBP* resulted in some non-specific binding. ApC/EBP isoform X1 is a truncated protein of ApC/EBP. Both ApC/EBP and its isoform X1 are functional transcription factors capable of binding to and activating enhancer response element (ERE) promoters. Lee et al. (2001) demonstrated a 16-fold increase in ERE-luciferase reporter expression in *Aplysia* neurons 24 h after DNA microinjection of *ApC/EBP* or its *isoform X1*. This indicated that ApC/EBP and its isoform X1 do not differ in function or effect. *ApC/EBP isoform X1* was used here as the proxy for *ApC/EBP*. In contrast, *Aplysia* has a single *teneurin* paralog, unlike *Drosophila*, with 2, *ten-a* and *ten-m* (Wides, 2019).

SYBR Green (Applied Biosystem *Power*SYBR Green PCR Master Mix) qPCR in a 96-well plate was conducted using a Stratagene thermocycler (Stratagene Mx3005P) under the following cycle parameters: 10 min initial denaturation at 95°C followed by 40 cycles of 30 s at 95°C, 1 min at 55°C, and 1 min at 72°C with fluorescence measured at the end of each cycle, and followed by a melt curve for 1 min at 95°C, 30 s at 55°C, and 30 s at 95°C. In this study, 2 μL of cDNA was used in each qPCR reaction which is equivalent to 10 ng of starting RNA. Gene specific primers for *teneurin*, i.e., forward primer, 5′-TCAACAGGATCCGAGTGGTCAGTA-3′, reverse primer, 5′TGCTACGACCCTCACGAGACA-3′ and *ApC/EBP isoform X1*: forward primer, 5′GCACAAACAAAGATCCCACGG-3′ reverse primer, 5′-CGGACGTGACGAGCTACTAC-3′, were used. Samples and standard curves were run in triplicate.

Standards for the absolute quantification of *ApC/EBP isoform X1* and *teneurin* transcripts were created by cloning PCR products using a TOPO II cloning kit (Thermo Fisher Scientific TOPO TA Cloning Kit, Dual Promoter, without competent cells) and then quantifying plasmid levels. Copy numbers were estimated by comparison with a standard curve (*teneurin m* = −3.36, efficiency = 98.4%; *ApC/EBP isoform X1 m* = −3.54, efficiency = 91.7%).

A time course study was conducted to pinpoint *teneurin’s* optimal gene expression window using age 13-month Aplysia from a separate cohort. LFI training was followed by trimmed buccal ganglia dissections at 1, 2, and 3 h after Day 2 recall testing. Two +%SAV animals for each timepoint were used to determine *teneurin* expression via qPCR.

### Statistical analysis

The Shapiro–Wilk test was used to test for normality. A Student’s *t*-test was used to determine significant differences in percent savings. The Wilcoxon signed-rank test was used to determine significant differences in TTIM within each age group, comparing TTIM on Day 1 to TTIM on Day 2, while multiple Wilcoxon rank-sum tests followed by a Bonferroni’s correction was used to determine significant differences in TTIM on Day 1 and on Day 2 between young and aged. Significant differences in bite frequency were determined through a chi-square test. Multiple chi-square tests followed by a Bonferroni’s correction were used to determine significant differences in bite frequency.

When comparing gene expression differences within an age class, a one-way ANOVA was used for *teneurin* expression, and a Kruskal–Wallis rank-sum test followed by a *post-hoc* Dunn test was used for *ApC/EBP isoform X1* expression. Multiple student’s *t*-tests followed by a Bonferroni’s multiple test correction were used to determine significant differences in *teneurin* expression across age groups. Multiple non-parametric Kruskal–Wallis rank-sum tests followed by a Bonferroni’s multiple test correction were used to determine significant differences in *ApC/EBP isoform X1* expression.

Pearson’s correlation coefficient was used for correlation analyses. Significant differences between months in reflex behaviors were determined by a Kruskal–Wallis rank-sum test and *post-hoc* Dunn test. Differences were considered significant at a *p*-value of ≤ 0.05 unless an adjustment was needed after performing a Bonferroni’s multiple test correction on the *p*-value.

## Results

### Age determination

**FIGURE 2:**
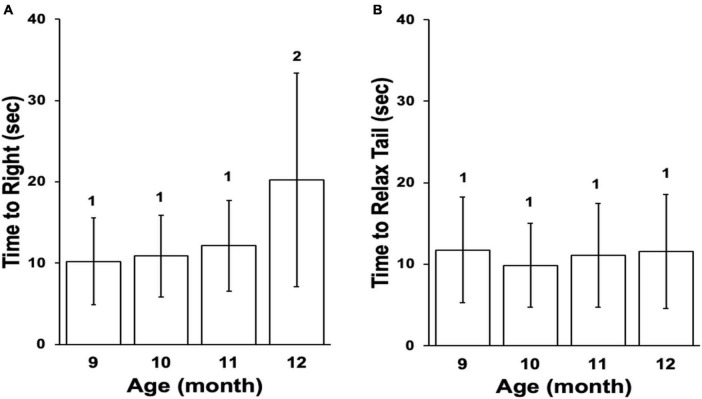
Reflex behaviors for the cohort. **(A)** Mean time to right in righting reflex, ± SD. **(B)** Mean time to relax tail in tail withdrawal reflex, ± SD. Differences in numbers above columns denote significance differences between months (Time to Right, Kruskal–Wallis rank sum test *p* ≤ 0.001; Dunn’s test 12 vs. 9 padj ≤ 0.001, 12 vs. 10 padj ≤ 0.01, 12 vs. 11 padj ≤ 0.05). The cohort entered stage Aged II at age 12 months based on the righting reflex.

Animal age in this study was determined by the chronological age as well as the significant decline in performance of reflex behaviors beginning at the age of 12 months (Figure 2). Our laboratory demonstrated that performance in these behaviors declines in a predictable manner with age (Kempsell and Fieber, 2014) and that declining performance is accompanied by a decline in excitability of PVC sensory neurons that control tail withdrawal, decreased short-term facilitation (STF) between tail sensory and motor neurons (Kempsell and Fieber, 2015), decreased expression of ionotropic glutamate receptor subunits (Greer et al., 2019), and other changes in gene expression that alter proteostasis and increase neuro-inflammation (Kron et al., 2020; Kron and Fieber, 2021).

### Behavioral results

**FIGURE 3:**
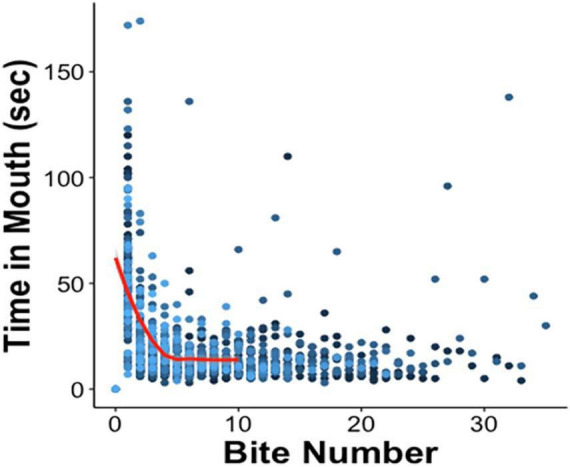
Negative correlation between time spent attempting to ingest the probe and the first five ingestion attempts on Day 1 [red curve; Pearson’s correlation coefficient r(230) = –0.57, *p* ≤ 0.001], after which the data begin to plateau as animals successfully complete training for that day. The amount of time the probe was held in the mouth was recorded for each bite. Each bite number along the *x*-axis records Day 1 tests of all 70 animals, in 70 different shades of blue.

Upon initial presentation of the probe in LFI, an animal advanced toward the netted algae, and once the probe touched the lips, it attempted to ingest it, likely due to hunger. It pulled the probe into its buccal cavity (biting) and then attempted to move the probe into its crop (swallow), which the probe was designed to thwart. Often during swallowing attempts, the animal’s radula could be felt scraping the probe as a vibration through the hemostat. Eventually, the animal pushed the probe out of its buccal cavity (exit). As training progressed, the duration of time the probe was held in the mouth decreased as interest in attempted consumption waned and then ceased altogether. Consistent with Susswein et al. (1986) findings, the relationship between attempts at ingestion on Day 1 and the time the probe remained in the mouth during that ingestion attempt were inversely correlated (Figure 3). Especially during the first five bites, the probe exited from the buccal cavity more quickly with each successive ingestion attempt.

**FIGURE 4:**
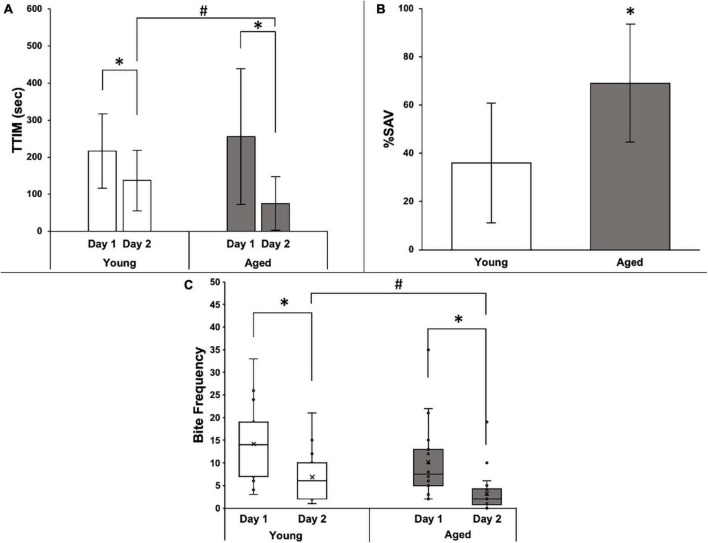
Behavioral data for 15 young and 22 aged sibling Aplysia. **(A)** Mean TTIM of young and aged sibling Aplysia, ± SD, on Day 1 and Day 2 training. *Denotes significant difference via Wilcoxon signed-rank (*p* ≤ 0.05: young *p* ≤ 0.001; aged *p* ≤ 0.001). ^#^Denotes a significant difference via the Wilcoxon rank-sum test and Bonferroni’s correction (padj ≤ 0.05). **(B)** Mean %SAV in young and aged sibling Aplysia, ± SD. *Denotes a significant difference via Student’s *t*-test (*p* ≤ 0.01). **(C)** Distribution of bite frequency of young and aged sibling Aplysia on Day 1 and Day 2 of LFI re-training. *Denotes significance difference via chi-square (*p* ≤ 0.001). ^#^Denotes significance difference via chi-square test and Bonferroni’s correction (padj ≤ 0.05).

Among +%SAV animals, there was no significant difference in the average TTIM on Day 1 between age groups; however, the mean Day 2 TTIM was significantly less than Day 1 in both young and aged animals (Figure 4A). Day 2 aged TTIM, in addition, was significantly less than young Day 2 TTIM, and thus, the aged animals that learned had significantly higher %SAV than their young siblings (Figure 4B). Biting followed a similar trend, with no significant difference between age groups in the distribution of biting occurrences on Day 1 and a significant reduction in biting occurrences on Day 2 in both young and aged animals (Figure 4C). Aged +%SAV siblings made significantly fewer biting attempts on Day 2 compared to young siblings, with an additional metric leading to higher %SAV in aged animals.

Five aged +%SAV Aplysia who qualified for Day 2 re-training showed no interest in the probe on Day 2. These animals were designated as 100% learners, with perfect recall that the probe was inedible on Day 2. They are included in the analyses of Figures 3, 4. An alternative explanation, however, is that they simply failed Day 2 training, a behavior that would have classified them as duds if it occurred on Day 1. This ambiguity warranted a separate analysis of the data in which these individuals were excluded. The results with their exclusion were largely unchanged, with the only difference being a loss in significance when comparing TTIM on Day 2 between the two +%SAV age groups (data not shown). Importantly, however, exclusion of the 100% learners resulted in a significantly higher %SAV compared to young siblings. Since the exclusion of 100% SAV individuals did not markedly alter the results, they were included in the final behavioral and gene expression analyses.

### Gene expression

**FIGURE 5:**
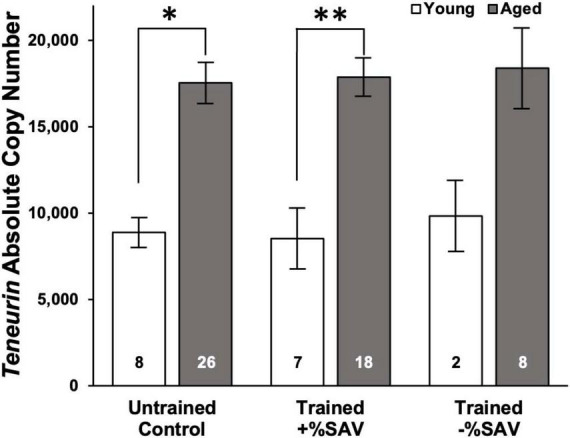
Mean *teneurin* copy number ± SE in young (white bars) and aged (gray bars) animals. ANOVA in young and aged animals, all categories, showed no significant differences. Multiple student’s *t*-tests with a Bonferroni’s correction were then used to test for significant differences between ages. *Denotes a significant difference via Student’s *t*-test (*p* ≤ 0.001). ^**^Denotes significance difference via Student’s *t*-test (*p* ≤ 0.001). Sample size noted within bars. *Teneurin* expression for young animals was quantified over 2 qPCR plates with the following standard curve slopes and primer efficiencies: Plate 1 *m* = –3.40, efficiency = 96.7%; Plate 2 *m* = –3.53, efficiency = 92.0%. *Teneurin* expression for aged animals was quantified over three qPCR plates with the following standard curve slopes and primer efficiencies: Plate 1 *m* = –3.46, efficiency = 94.5%; Plate 2 *m* = –3.56, efficiency = 90.8%; Plate 3 *m* = –3.47, efficiency = 94.3%.

*Teneurin* expression was significantly elevated in aged animals in untrained and trained +%SAV categories compared to their young sibling counterparts (Figure 5) but did not differ among the aged categories. Similarly, the young animals in all training categories showed no significant differences in *teneurin* expression.

The *teneurin* expression was not correlated to individual animals’ %SAV (data not shown). A time course experiment on aged animals to determine whether the time of sampling after LFI training affected *teneurin* expression showed that time of sampling at 1, 2, or 3 h after recall testing did not significantly influence *teneurin* gene expression (data not shown).

**FIGURE 6:**
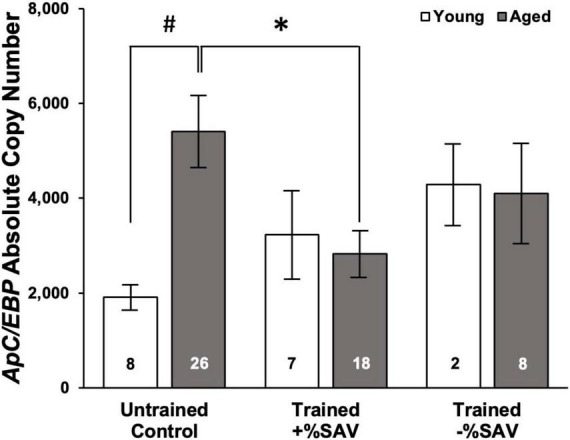
Mean *ApC/EBP isoform X1* copy number, ± SE in young (white bars) and aged (gray bars) animals. Multiple Kruskal–Wallis rank sum tests with a Bonferroni’s correction were used to test for significant differences. *Denotes a significant difference (*p* ≤ 0.05) via Kruskal–Wallis rank sum test (padj ≤ 0.05 *via* Dunn’s test). ^#^Denotes significance difference via Kruskal–Wallis rank sum test (*p* ≤ 0.01). *ApC/EBP isoform X1* expression for both young and aged animals was quantified over four qPCR plates with the following standard curve slopes and primer efficiencies: Plate 1 *m* = –3.72, efficiency = 85.8%; Plate 2 *m* = –3.61, efficiency = 89.2%; Plate 3 *m* = –3.66, efficiency = 87.5%; Plate 4 *m* = –3.54, efficiency = 91.7%.

**FIGURE 7:**
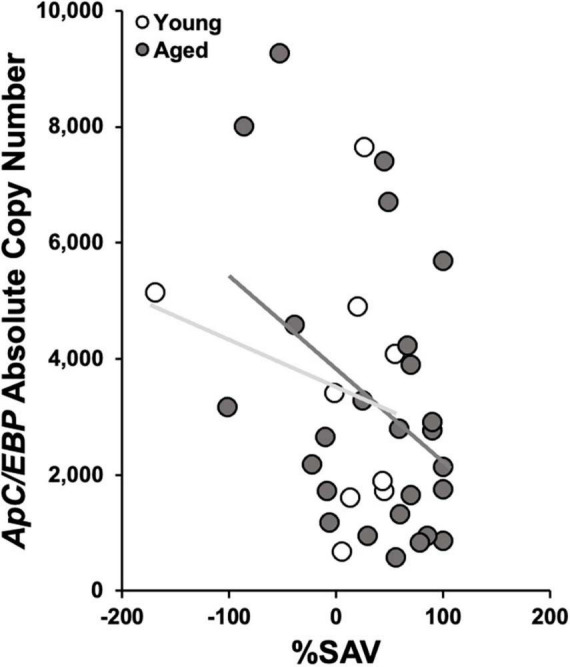
*ApC/EBP isoform X1* copy number as a function of %SAV. Aged animal expression was negatively correlated to performance via Pearson’s correlation coefficient [gray; r(24) = –0.39, *p* ≤ 0.05; regression line in dark gray]. No significant correlation was found in the young samples (white; regression line light gray).

**FIGURE 8:**
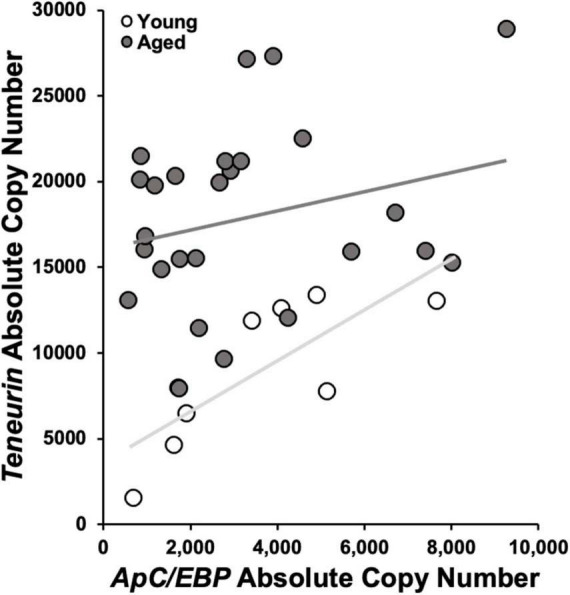
*Teneurin* copy number as a function of *ApC/EBP isoform X1* copy number. Young animal expression was significantly positively correlated via Pearson’s correlation coefficient [white; r(7) = 0.78, *p* ≤ 0.05]. No significant correlation was found in aged samples (gray). Regression lines as in Figure 7.

In aged animals, there was a significant reduction in *ApC/EBP isoform X1* expression in +%SAV animals compared to untrained controls (Figure 6). Expression was significantly elevated in aged untrained controls compared to their younger siblings. Young animals in all training categories showed no significant differences in expression. Individual animals’ *ApC/EBP isoform X1* expression and %SAV showed a negative correlation only in the aged animals (Figure 7). Finally, *ApC/EBP isoform X1* expression and *teneurin* expression were significantly positively correlated only in the young animals (Figure 8).

## Discussion

Young and aged Aplysia both demonstrated learning and recall in LFI. Some failed the Day 1 learning protocol by not attaining the TTIM threshold of 100 s common to these studies (Levitan et al., 2012; Tam et al., 2020), while others failed Day 2 recall testing when their TTIM was greater than that on Day 1. Aged animals failed at a greater proportion on both Day 1 and Day 2. This resulted in more aged animals being tested to achieve a sample size of good performers (+%SAV) in age to compare to young animals. It was particularly important to have a standardized learning threshold for gene expression analysis linked to learning performance. The necessity to test more aged animals is a common dynamic in aged learning (Gage et al., 1984; Lee et al., 1994; Maliković et al., 2019); however, it may have resulted in selection bias in its focus solely on the animals from each age group that learned on Day 1 and demonstrated recall on Day 2. The aged animals that learned had significantly lower TTIM on Day 2, resulting in significantly higher +%SAV than their younger siblings.

There have been few studies that demonstrated increased cognitive performance in age, always specific to operant conditioning (Samson et al., 2014). LFI, as an operant paradigm (Michel et al., 2011), is consistent with this pattern. In contrast, and more commonly reported, there are many other studies that assert cognition declines with age, including spatial learning in rats (Adams et al., 2008); spatial, visual, and temporal learning in fish (Yu et al., 2006; Terzibasi et al., 2008); sensitization and habituation in *Aplysia* (Kempsell and Fieber, 2015, 2016); electric shock avoidance, locomotion, geotaxis, and phototaxis in *Drosophila* (Simon et al., 2006); and isothermal tracking in *C. elegans* (Murakami and Murakami, 2005).

In operant conditioning, organisms will learn to change behavior to receive rewards or avoid punishments (Skinner, 1938). In LFI, the negative reinforcement (Michel et al., 2011) of inedibility, culminating in ceasing attempts to ingest the probe (Schwarz and Susswein, 1986), was better avoided in aged animals compared to their younger siblings. This is similar to a phenomenon observed in humans who commonly shift away from reward-based and toward loss-avoidance operant learning during aging (Freund and Ebner, 2005; Frank and Kong, 2008; Horn and Freund, 2021).

Another interpretation is that aged *Aplysia* are more selective eaters than their younger siblings. Wild *A. californica* has few natural predators (Moroz, 2011) and an abundant food supply, which allows it to be a preferential eater (Kupfermann and Carew, 1974). Preferential eating has been observed in the laboratory (Carefoot, 1967), and firsthand while rearing *Aplysia* in this study, where hungry animals refused *U. lactuca*, fasting until the preferred *A. subulata* was offered. Aged *Aplysia* may have been faster to refuse the *Ulva* food probe, potentially waiting for the preferred *Agardhiella* to be offered.

A plausible interpretation of aged animal performance in LFI is that aged *Aplysia* that learned did not necessarily learn better in LFI, but rather they remembered their training better on Day 2, signifying that a memory was formed. This would place Day 2 of LFI not as a day of re-training but as a test of the animal’s LTM of Day 1’s training (Schwarz et al., 1991; Botzer et al., 1998; Katzoff et al., 2002; Michel et al., 2011; Krishnan et al., 2016). Trained animals on Day 1 learned to recognize the inedible probe in an approximately equal time and number of ingestion attempts, and it was only on Day 2 that age benefitted recognition of the probe as inedible. This behavior was also observed in aged rats during extinction training, in which Day 1 performance was no different, but on Day 2, aged rats performed significantly better than their younger counterparts (Samson et al., 2014). In this view, data suggest that the aged animals that learned had a better ability to store and recall LTM of LFI than younger siblings.

*ApC/EBP isoform X1* expression was either unchanged (young animals) or decreased (aged animals) after 2 days of LFI training in trimmed buccal ganglia neurons. This was unexpected; *ApC/EBP’s* role in LFI necessitates that it rises in expression to facilitate long-term facilitation (LTF). The explanation may lie in timing of the qPCR analysis of *ApC/EBP isoform X1* transcripts as well as the trimmed buccal ganglia sample preparation.

Learning food is inedible training was shown to induce *ApC/EBP* expression 15 min, 1 h, and 2 h after Day 1 LFI training in isolated buccal S cluster (BSC) sensory neurons of the buccal ganglia but not elsewhere (Levitan et al., 2008, 2012; Briskin-Luchinsky et al., 2018), implying that the memory for LFI was stored in the BSC. It was later discovered that LFI facilitates functional rewiring between BSC neuronal processes and their postsynaptic motoneurons, resulting in changes in synaptic strength and number of synaptic connections and culminating in the flipping of postsynaptic potentials between excitatory and inhibitory (Tam et al., 2020). However, this still did not explain the physiological changes that accompany LFI, suggesting other synaptic modifications must be involved.

For example, cerebral-buccal interneurons (CBIs) of the cerebral ganglia were shown to decrease their responsiveness to the neurotransmitter acetylcholine (ACh) after LFI (McManus et al., 2019). ACh, released from sensory afferent neurons after the animal is stimulated by food, depolarized CBIs (Susswein et al., 1996), which then activates central pattern generators of the buccal ganglia that control the animal’s feeding reflexes (Hurwitz et al., 2003; Jing et al., 2003). Unresponsiveness in CBI resulted in fewer biting responses and a shortened duration of ingestion attempts (McManus et al., 2019), illustrating that memory for LFI is distributed among multiple sites within the neural circuit controlling the reflex and not confined to BSC neurons as previously thought.

Here, BSC neurons were removed from the buccal ganglia to enhance the detection of *ApC/EBP isoform X1* and *teneurin* expression changes in a subset of inter- and motoneurons of the buccal ganglia, suggested to be important in the freshwater pond snail *Lymnaea stagnalis* (Perry et al., 1998; Hatakeyama et al., 2006), where *C/EBP* mRNA expression decreased after conditioned taste aversion (CTA) training and C/EBP protein production and phosphorylation increased. This suggested that training rapidly induced the translation and then immediate degradation of available *C/EBP* transcripts in postsynaptic motoneurons, a pattern that may have played out here in trimmed buccal ganglia samples after LFI.

One of the outcomes of LFI is a reduction in radula protractions. Changes in B31/B32 activity were hypothesized to be implicated and to experience changes in expression of *ApC/EBP isoform X1*. This either was not observed or not measured accurately as trimmed buccal ganglia samples included other inter- and motoneurons along with B31/B32. While the goal of trimming the buccal ganglia was to boost the signal coming from B31/B32 by eliminating signals from the BSCs along with a great proportion of inter- and motoneurons that lie superior to the BSCs, B31/B32 may have remained only minor contributors to the overall expression signal coming from the sample. Levitan et al. (2012) hypothesized that the inclusion of superfluous cells retards the detection of even large changes in *ApC/EBP* expression. It may not be possible to accurately infer LFI-related changes in *ApC/EBP isoform X1* in this study for this reason.

Another possible reason for failure to detect changes in *ApC/EBP isoform X1* expression in this study is sampling may have occurred outside the appropriate time window. Levitan et al. (2012) noticed that *ApC/EBP* expression changes were undetectable in BSCs 24 h after LFI. Therefore, *ApC/EBP isoform X1*-induced memory consolidation may have already taken place by the start of Day 2 recall testing, and gene expression levels returned to normal. Detectable after Day 2 of LFI would instead be genes involved in the reconsolidation of LFI memory, and it is possible that *ApC/EBP isoform X1* plays no role in this process (Taubenfeld et al., 2001).

A significantly higher *ApC/EBP isoform X1* expression was noted in aged untrained *Aplysia* compared to young siblings. While in the absence of a learning component, this increase is common in neurons as animals age, for example, human and rat (Wang et al., 2018), and has been suggested to reflect a role in inflammation, the overexpression of *ApC/EBP* by DNA microinjection has also been shown to increase the efficiency of LTF induction after a single pulse of 5-HT (Lee et al., 2001), demonstrating that the more *ApC/EBP* available at the time of training, the faster LTF occurs and in response to fewer repeated stimuli. The significant reduction in *ApC/EBP isoform X1* in aged trained *Aplysia* compared to their untrained siblings and the negative correlation of *ApC/EBP isoform X1* to increased +%SAV suggest that the neurons are rapidly translating ApC/EBP isoform X1 protein from the larger reservoir of *ApC/EBP isoform X1* mRNA in aged neurons, *ApC/EBP isoform X1* mRNA is then being rapidly degraded as was shown in *L. stagnalis* after CTA (Hatakeyama et al., 2006), and this process may be aiding the performance of LFI in aged *Aplysia*.

*Teneurin* expression was elevated in aged animals, yet after testing for recall of LFI, there was no effect on its expression in either young or aged animals. Its failure to increase may indicate that *teneurin* expression is not affected by learning. Gene expression results did not corroborate the hypothesis that teneurin is involved in learning-induced synaptic modifications because changes in *teneurin* expression in +%SAV animals were not detected. This may be due to the goal of testing gene expression during the reconsolidation of memory and not during the consolidation process, which can initiate different transcriptional pathways (Hertzen and Giese, 2005), resulting in either enhancement or reduction of the memory (Lee et al., 2017).

Much similar to the results for *ApC/EBP isoform X1*, qPCR analysis may have taken place long after *teneurin* expression was expected to be affected by learning. Samples were taken 2 h after animals were showing signs of LTM from the previous day’s training. This indicated that synaptic connections had already been altered during the consolidation of LFI. If no longer needed for the consolidation of memory, *teneurin* expression levels may have receded back to normal. As for *ApC/EBP isoform X1*, dilution also was possible, in a preparation designed to bolster the contribution of neurons B31/B32 which are small and anonymous absent electrophysiological recording (Susswein and Byrne, 1988; Dembrow et al., 2004). It is therefore not possible to accurately conclude that teneurin is not involved in memory consolidation based on these results.

In the *Drosophila* mutant *central body defect (cbd)* which is deficient in teneurin isoform, *ten-a*, development in regions of the brain important to visual pattern memory is disrupted (Pan et al., 2009; Cheng et al., 2013), and olfactory learning is impacted (Heisenberg et al., 1985). The overexpression of *ten-a* restores normal development and learning, while downregulation through RNAi disrupts them (Cheng et al., 2013). A similar causal link between *teneurin* expression and learning in Aplysia remains undemonstrated; however, higher *teneurin* levels may result in better learners or a stronger consolidation of memory. Aged animals that learned in LFI had elevated *teneurin* levels and were significantly better performers.

## Conclusion

Young and aged *Aplysia* that demonstrated learning and recall in LFI were compared for behavioral and transcriptional differences. Aged *Aplysia* selected for their success in the protocol, which occurred less frequently in age than in youth, demonstrated recall superior to their younger siblings. This coincided with differences in gene expression underlying the feeding reflex between the two age groups. Neurons from naive aged *Aplysia* showed increased transcript levels of two genes that are believed to be involved in learning, which may have benefitted the aged *Aplysia* that learned in their increased ability to recall LFI. It is also possible that aged animals that learned in the protocol experienced a shift in learning performance from reward-based to loss avoidance captured in the negative operant conditioning of LFI.

## Data availability statement

The original contributions presented in this study are included in the article/supplementary material, further inquiries can be directed to the corresponding author.

## Ethics statement

Ethical review and approval was not required for the study of animals in accordance with the local legislation and institutional requirements.

## Author contributions

ER and LF conceived the research executed by ER and summarized the data. ER executed statistical analysis. Both authors co-wrote the manuscript and approved the submitted version.
